# Molecular Evolutionary and Epidemiological Dynamics of Genotypes 1G and 2B of Rubella Virus

**DOI:** 10.1371/journal.pone.0110082

**Published:** 2014-10-17

**Authors:** Abinash Padhi, Li Ma

**Affiliations:** Department of Animal and Avian Sciences, University of Maryland, College Park, Maryland, United States of America; University of Florida, United States of America

## Abstract

Rubella Virus (RV), which causes measles-like rashes in children, puts millions of infants at risk of congenital defects across the globe. Employing phylogenetic approaches to the whole genome sequence data and E1 glycoprotein sequence data, the present study reports the substitution rates and dates of emergence of all thirteen previously described rubella genotypes, and gains important insights into the epidemiological dynamics of two geographically widely distributed genotypes 1G and 2B. The overall nucleotide substitution rate of this non-vector-borne RV is in the order of 10^−3^ substitutions/site/year, which is considerably higher than the substitution rates previously reported for the vector-borne alphaviruses within the same family. Currently circulating strains of RV share a common ancestor that existed within the last 150 years, with 95% Highest Posterior Density values ranging from 1868 to 1926 AD. Viral strains within the respective genotypes began diverging between the year 1930 s and 1980 s. Both genotype 1G and 2B have shown a decline in effective number of infections since 1990 s, a period during which mass immunization programs against RV were adapted across the globe. Although both genotypes showed some extent of spatial genetic structuring, the analyses also depicted an inter-continental viral dispersal. Such a viral dispersal pattern could be related to the migration of infected individuals across the regions coupled with a low coverage of MMR vaccination.

## Introduction

The increasingly intercontinental movements of people, commodities, and trade undoubtedly have a significant impact on the economic growth of every nation; however, these movements could also make any nation susceptible to infectious diseases [1]. In today’s interconnected world, if infectious viral pathogens are still spreading in parts of the world, reappearance of these viral pathogens will be possible in other regions that once declared complete elimination [1]. For instance, in early 2000’s, although the rubella virus (RV) was declared to be completely eliminated from the United States, there are increasing evidence of importation of viral genotypes from Asian and South American countries [2]–[5]. Complete elimination of RV genotypes through the national immunization programs and effective surveillance from every part of the world are, therefore, crucial in order to avoid the spread of RV.

RV, a positive single-stranded RNA virus of the genus Rubivirus in the Togaviridae family, causes measles-like mild rashes in children. In non-immunized women, it can severely affect the fetus during early pregnancy, resulting in miscarriage, fetal death, or birth defects such as congenital rubella syndrome (CRS), which includes heart disease, blindness and deafness [6]–[10]. According to the World Health Organization (WHO), approximately 112,000 babies around the world are born with CRS every year, thus making rubella a leading cause of preventable congenital defects [8]–[10]. The use of rubella vaccine began in 1969 [11]. Since 1970 s, combined formulations of RV vaccines with measles (MR vaccine) or with measles and mumps (MMR vaccine) have been administered [8]–[11]. Although the developed world has succeeded in eliminating RV and CRS through mass vaccination, the poor coverage of vaccination in many countries in the Southeast Asia, South America and Africa regions remains a global concern [2], [8]–[10], [12]–[21].

The Rubella viral genome is approximately 9.8 kb of length that encodes two nonstructural polypeptides (P150 and P90) and three structural polypeptides (C, E2, and E1) [21]. A 739-bp region within the E1 glycoprotein, which contains important functional domains including hemagglutination-inhibiting and -neutralizing epitopes and antigenic sites [6], [22], has been designated to be the minimum acceptable sequence window for assigning genotypes of RV [23]. Phylogenetic analyses based on the 739-bp sequence revealed the existence of two distinct clades (clade 1 and clade 2) [2]. While clade 1 is comprised of 10 genotypes, including 9 recognized (1B, 1C, 1D, 1E, 1F, 1G, 1H, 1I, and 1J) and 1 provisional (1a), clade 2 includes three genotypes (2A, 2B, and 2C) [24]. Of these 13 genotypes, 10 genotypes exhibit a restricted geographic distribution, whereas 3 genotypes, 1E, 1G and 2B, have a wide geographic distribution [2]. While isolates belonging to genotype 2B are circulating across the Asia, Americas, and Europe, 1G isolates appear to be widely spread in two continents, mostly in Africa and Europe [2]. The differences in their distribution patterns prompted us to hypothesize that each genotype experiences a unique evolutionary history, rate of evolution, and selection pressure.

Despite the mass vaccination efforts against RV, the wide geographical spread of these genotypes remains a global challenge. Therefore, it is imperative to uncover details of the genetic and epidemiological dynamics of these viral genotypes. Knowing the patterns of genetic variations over time of each viral genotype would allow us to better understand the effects of vaccination and host movements on viral diversity as well as to predict future disease outbreaks. Employing phylogenetic analyses to the whole genome sequence data and the E1 gene sequence data, in this study we report the estimated nucleotide substitution rates and dates of emergence of different genotypes of RV and reveal epidemiological dynamics of two widely distributed genotypes, 1G and 2B.

## Materials and Methods

To estimate the substitution rate and date of emergence of different rubella viral genotypes, and to gain insights into the epidemiology of genotypes 1G and 2B, dated nucleotide sequences (739 bp in length) representing all the 13 RV genotypes were retrieved from GenBank [25], together with the country of origin of the isolates. A total of 27 complete genome sequences that comprised of 10 genotypes representing both clade 1 and clade 2 were also retrieved from GenBank. We estimated the substitution rate and date of emergence using the 739-bp sequences of the E1 glycoprotein of RV. Evolutionary parameters were also estimated from the complete rubella viral genome (9762 bp), structural protein (SP; 3192 bp) as well as from the nonstructural (NSP; 6351 bp) genomic regions. The number of sequences analyzed in each dataset is listed in Table 1. GenBank accession numbers of the sequences used in the analyses are shown in Appendix S1. Sequences were aligned using the clustalW algorithm implemented in MEGA ver 4 [26]. The appropriate model of nucleotide substitutions for each data set was selected by the Bayesian Information Criterion (BIC) implemented in jModelTest2 [27], [28]. The PhyML ver 2.44 [28] program was used to reconstruct the maximum-likelihood (ML) phylogenies for genotypes 1G and 2B under the appropriate model of nucleotide substitutions. Using the same program, nodal supports were estimated with 1000 bootstrap replicates. We employed a Bayesian Markov Chain Monte Carlo (MCMC) approach to estimate the overall substitution rate (measured in the unit of substitutions per site per year) and the time to the most recent common ancestor (TMRCA) under a relaxed clock model with an uncorrelated lognormal distribution and under a strict clock model imposing the rate consistency, with different coalescent priors (constant population size and Bayesian skyline plot: BSP) implemented in BEAST ver. 1.8.0 [29]. Phylogenies were evaluated using a chain length of 20–50 million states (varied with the number of sequences) under Tamura-Nei (TrN) nucleotide substitution models with a gamma distribution shape parameter. In each case, MCMC chains were run for sufficient time to achieve convergence. Proper mixing of the MCMC was evaluated by calculating the effective sampling size (ESS) for each parameter [29]. All ESS values were >200, indicating sufficient mixing of the Markov chain. Uncertainty in the data was measured by 95% highest-posterior density (HPD) intervals. Convergence of trees was checked using Tracer v1.5 (available at: http://beast.bio.ed.ac.uk/Tracer). The inferred trees were visualized using FigTree ver. 1.3.1 (available at: http://tree.bio.ed.ac.uk/software/figtree/). In addition to constant population size and BSP, we also used the Gaussian Markov Random Field (GMRF) Bayesian skyride [30] coalescent prior to infer the past population dynamics of 1G and 2B genotypes. For BSP coalescence prior, the group sizes for 1G and 2B were set to 15 and 24, respectively. Different clock models and best-fit demographic models were evaluated by a Bayes factor (BF), which is the difference in the marginal log likelihood of two models [31]. Evidence against the null model, the model with a lower marginal likelihood, is indicated by either 2ln(BF) >3 (positive evidence) or 2ln(BF) >10 (strong evidence) [32]. The clock-like behavior was also assessed from the coefficient of variation (CoV) statistic [33]. If the posterior distribution of CoV does not encompass zero, it indicates that the relaxed clock model provides a better fit to the data than the strict clock model.

**Table 1:** Estimates of mean substitution rates (×10^−3^ nucleotide substitutions per site per year), TMRCAs (in year) and the dN/dS for genotypes 1G, 2B and for the pooled dataset.

Genotype	n	Genomic region	Overall dN/dS	Coalescentprior	Clockmodel	Marginal loglikelihood	BayesFactor	Mean substitutionrate (95% HPD)	TMRCA (95% HPD)	Relaxed clock CoV
10genotypes	27	Whole genome(9762-bp)		Constant	Strict	−38873.2		0.78 (0.70 −0.86)	1900 (1892 −1908)	NA
					Relax	−38798.7	148.938	0.81 (0.59 −1.04)	1898 (1863 −1925)	0.35 (0.23 −0.50)
				**BSP**	Strict	−38873		0.78 (0.70 −0.85)	1900 (1892 −1908)	NA
					**Relax**	−**38798.5**	**149.118**	**0.79 (0.57 −1.04)**	**1899 (1863 −1929)**	**0.35 (0.24 −0.49)**
	27	SP (3192-bp)	0.05 (0.044 −0.058)	**Constant**	Strict	−12895.6		0.68 (0.56 −0.80)	1890 (1871 −1906)	NA
					**Relax**	−**12880.8**	**29.6**	**0.75 (0.58 −0.96)**	**1897 (1869 −1922)**	**0.26 (0.15 −0.42)**
				BSP	Strict	−12895.4		0.66 (0.54 −0.78)	1889 (1869 −1905)	NA
					Relax	−12881	28.802	0.73 (0.52 −0.93)	1897 (1865 −1923)	0.26 (0.14 −0.40)
	27	NSP (6351-bp)	0.052 (0.047 −0.057)	**Constant**	Strict	−25043.1		0.85 (0.76 −0.94)	1909 (1901 −1917)	NA
					**Relax**	−**24993.3**	**99.706**	**0.87 (0.67 −1.11)**	**1905 (1874 −1930)**	**0.34 (0.22 −0.49)**
				BSP	Strict	−25043.5		0.85 (0.75 −0.94)	1909 (1901 −1917)	NA
					Relax	−24994.1	98.764	0.88 (0.66 −1.10)	1909 (1879 −1931)	0.34 (0.22 −0.48)
All	115	E1 (739-bp)	0.020 (0.015 −0.028)	Constant	Strict	−5395.82		0.91 (0.75 −1.10)	1900 (1880 −1919)	NA
					Relax	−5376.22	39.186	0.98 (0.77 −1.20)	1900 (1868 −1926)	0.40 (0.23 −0.59)
				**BSP**	Strict	−5389.89		0.86 (0.68 −1.04)	1900 (1878 −1919)	NA
					**Relax**	−**5375.23**	**29.31**	**0.93 (0.73 −1.15)**	**1904 (1875 −1929)**	**0.36 (0.15 −0.55)**
1G	87	E1 (739-bp)	0.043 (0.028 −0.063)	Constant	Strict	−2825.24		1.23 (0.99 −1.49)	1982 (1977 −1986)	NA
					Relax	−2822.1	6.294	1.25 (1.00 −1.53)	1982 (1976 −1987)	0.24 (0.00006 −0.57)
				**BSP**	Strict	−2730.58		1.47 (1.15 −1.84)	1984 (1979 −1988)	NA
					**Relax**	−**2729.08**	**3.01**	**1.49 (1.14 −1.87)**	**1984 (1979 −1988)**	**0.16 (0.00002 −0.44)**
				GMRF	Strict	−2820.66		1.27 (1.03 −1.51)	1986 (1984 −1989)	NA
					Relax	−2818.92	3.476	1.27 (1.04 −1.54)	1986 (1984 −1989)	0.15 (0.00003 −0.41)
2B	168	E1 (739-bp)	0.041 (0.028 −0.059)	Constant	Strict	−3846.25		1.83 (1.46 −2.22)	1964 (1958 −1968)	NA
					Relax	−3799.44	93.632	2.02 (1.57 −2.49)	1961 (1943 −1968)	0.97 (0.62 −1.38)
				BSP	Strict	−3847.28		1.86 (1.49 −2.25)	1965 (1959 −1960)	NA
					Relax	−3801.24	92.078	1.99 (1.55 −2.44)	1964 (1949 −1968)	0.94 (0.64 −1.31)
				**GMRF**	Strict	−3845.18		1.57 (1.27 −1.88)	1968 (1965 −1968)	NA
					**Relax**	−**3799.16**	**92.04**	**1.60 (1.20 −2.10)**	**1968 (1965 −1968)**	**0.86 (0.53 −1.23)**

Best-fit models with the highest marginal log likelihood score for respective data sets are in bold.

n: Number of sequences; SP:Structural protein; NSP:Nonstructural protein; HPD: Highest Posterior Density; TMRCA: Time to the Most Recent Common Ancestor; CoV: Coefficinet of Variation; NA: Not Applicable; dN: Nonsynonymous substitution; dS: Synonymous substitution.

Taking into account the country of origin of the isolates from six regions (African: AFR; Americas: AMR; South-East Asia: SEAR; European: EUR; Eastern Mediterranean: EMR; and Western Pacific: WPR) that were previously defined by WHO [10], viral isolates in respective genotypes (1G and 2B) were assigned to respective WHO defined regions. ARLEQUIN version 3.5 [34] was used to estimate the pair-wise *F*st among the regions and significance testing (*p*<0.01) was performed based on 10,000 simulations.

We estimated the overall rate of nonsynonyomous (dN) to synonymous (dS) substitutions to identify positively selected codons in each data set using the Single Likelihood Ancestor Counting (SLAC), Fixed Effects Likelihood (FEL), and Fast Unconstrained Bayesian Approximation (FUBAR) methods implemented in the Datamonkey web server (http://www.datamonkey.org/) [35]–[37].

## Results

**Figure 1:**
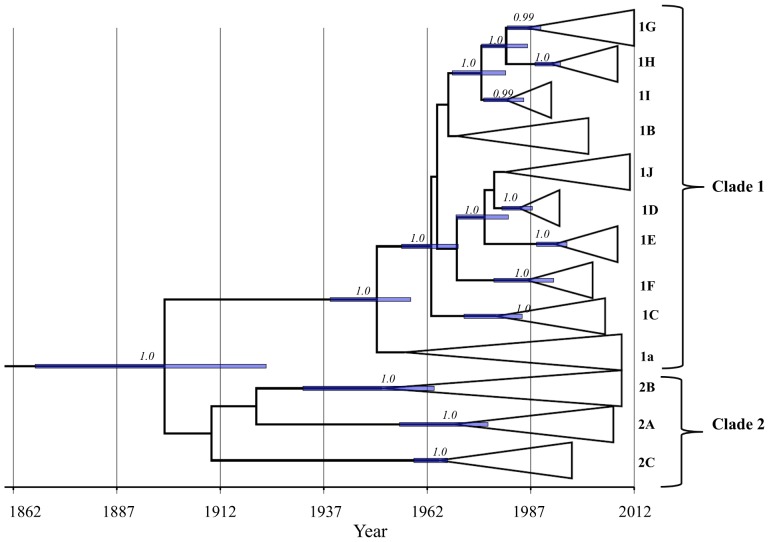
Maximum clade credibility (MCC) tree depicting TMRCA estimates of respective genotypes. Posterior probability of each node is shown. The horizontal bar at each node is the 95% HPD interval for the TMRCA of the respective node. Time-scale (in year) is shown at the bottom of the tree.

**Figure 2:**
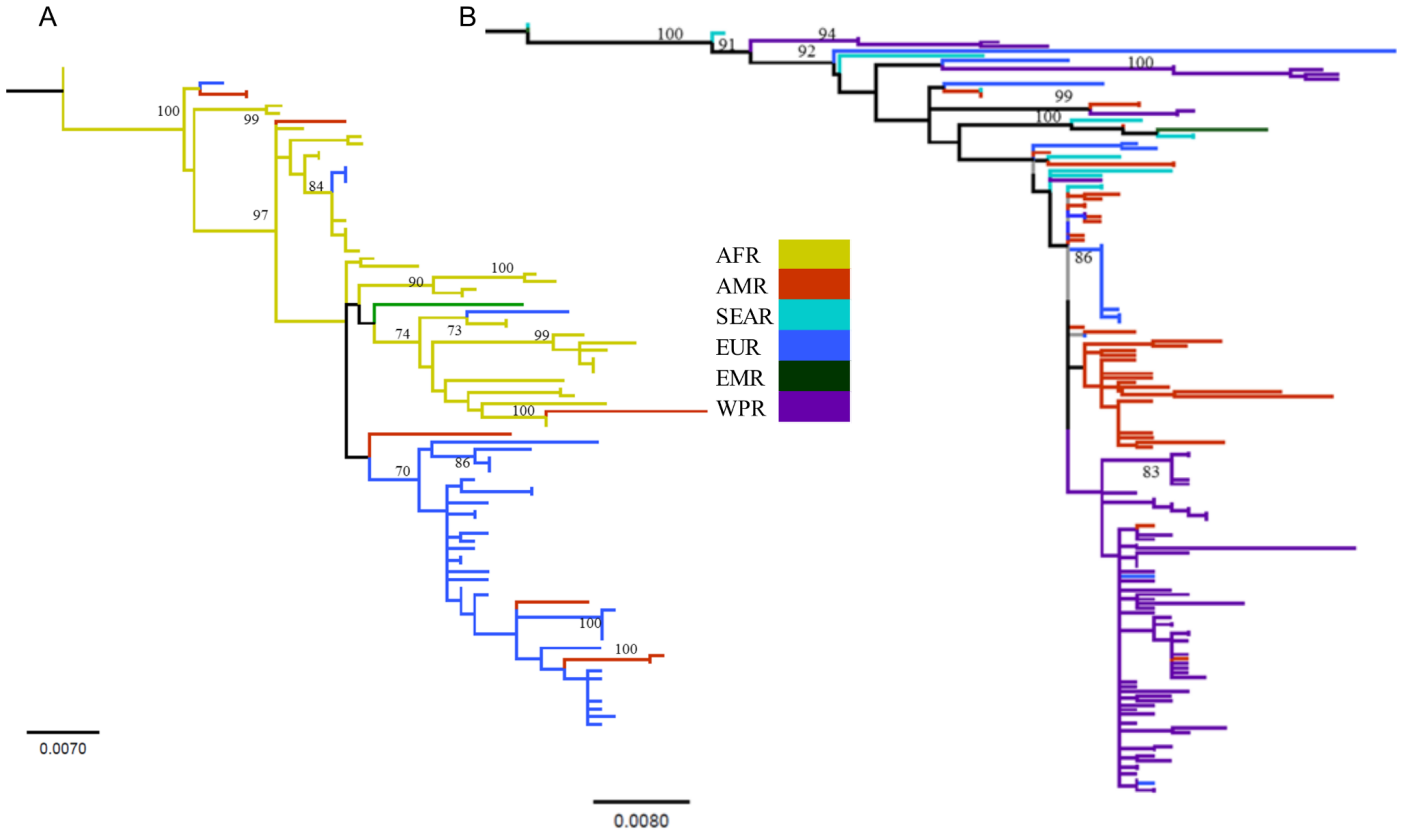
Maximum likelihood trees depicting phylogenetic clustering of the isolates from different regions. (**A**) Genotype 1G and (**B**) genotype 2B. Isolates from respective regions are color coded. Although both trees showed multiple genetic clustering, none of the clusters are supported by strong bootstrap support (>70). AFR: African region, AMR: Americas, SEAR: South-East Asia, EUR: European, EMR: Eastern Mediterranean, and WPR: Western Pacific region.

**Figure 3:**
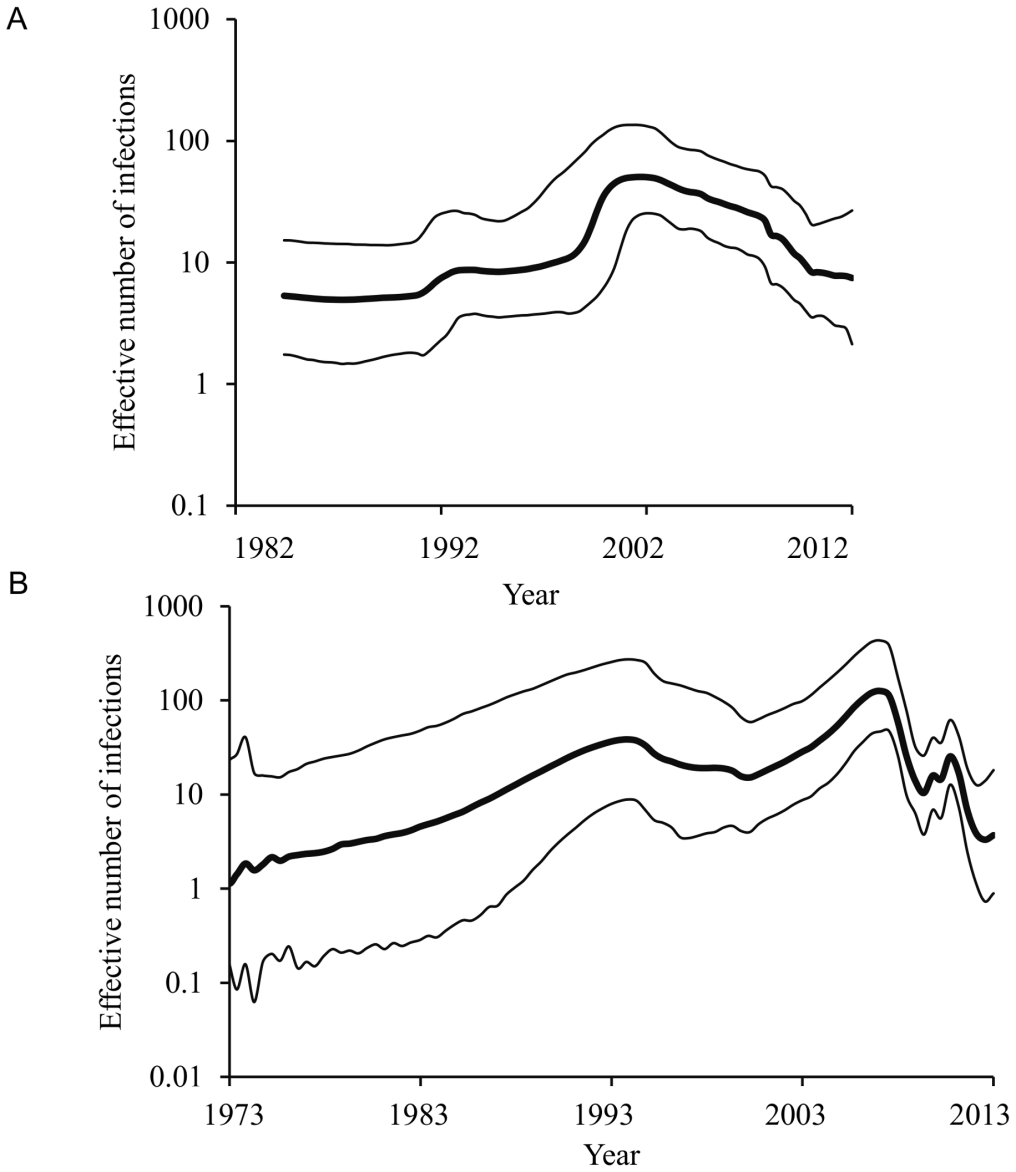
The best-fit Bayesian plots of effective number of infections over time for genotypes 1G and 2B. (**A**) Bayesian skyline plot of 1G and (**B**) GMRF Bayesian Skyride plot of 2B. The x-axis is in units of year, and the y-axis represents the effective number of infections. The thick solid black line is the median estimate, and the dotted lines show the upper and lower bounds of the 95% HPD interval.

By employing the Bayesian coalescent and ML-based phylogenetic approaches to the whole genome sequence data and 739-bp E1 nucleotide sequence data, the present study reports the estimated rate and date of emergence of each rubella viral genotype (Fig. 1; Table 1) and infers the population dynamics of genotypes 1G and 2B (Fig. 2A & B; 3A & B; Table 1). Two molecular clock models (strict and relaxed) with different coalescent priors were fitted to infer the rate and date of emergence of rubella viral genotypes. The Bayes factor (BF) using either a constant population size, BSP, or GMRF skyride coalescent prior favored the relaxed over the strict clock model (Table 1). With exception of 1G, under the relaxed molecular clock, the CoV for all the data sets did not encompass zero, indicating a large scale rate variation across the lineages (Table 1). We found a large degree of overlap (within the 95%HPD) in the evolutionary rate estimated from the whole genome, SP, NSP, as well as from the partial E1 nucleotide sequences (739-bp) (Table 1). Based on the relaxed clock assumption, the estimated evolutionary rates of RV inferred from the whole genome, SP, NSP and partial E1 nucleotide sequences are all within the range of 0.57–1.15×10^−3^ substitutions per site per year. According to our estimate, the currently circulating strains of RV share a common ancestor that existed within the last 150 years, with 95% HPD values ranging from 1863 to 1930 AD (Table 1; Fig. 1). While clade 1, which is comprised of ten genotypes, appeared to be monophyletic, clustering of the three genotypes that were previously designated to be within clade 2 is not supported (posterior probability <0.99). However, clustering of the RV strains within their respective genotypes in clade 2 is strongly supported with a posterior probability of 1.0, and the cluster began diverging between the year 1940 s and 1980 s (Fig. 1). All the 10 genotypes within clade 1 were descended from their most recent common ancestor between the year 1930 s and 1980 s (Fig. 1).

To gain insights into the emergence of RV, we investigated the population dynamics of genotypes 1G and 2B with a comparative approach. The mean TMRCAs of the genotypes 1G and 2B were estimated to be 1984 (95% HPD: 1979–1988 AD) and 1964 (95% HPD: 1943–1968 AD), respectively. ML-based phylogenetic analyses have revealed the existence of multiple genetic clusters in both genotypes 1G and 2B, yet with a weak bootstrap support (Fig. 2A and B). Although each genotype depicted an extent of spatial genetic clustering (Table 2 and 3), viral dispersals across the regions are also pronounced (Fig. 2A & B). Concurrently, pair-wise *F*st estimates for the respective genotypes indicate significant genetic differentiation (*p*<0.01) among the regions (Table 2 and 3). Within genotype 1G, AMR and AFR regions did not show significant genetic differentiation (Table 2), indicating a potential viral gene flow between these two regions. Given the high mutation rates of RV (Table 1), the lack of existence of the geography-based monophyletic lineages of the isolates may indicate increasingly international movements of unvaccinated individuals. Due to the limited sample size, however, it is difficult to infer the global phylogeographic patterns. Therefore, to infer the global phylogeographic pattern of each genotype, future studies should be carried out with a larger sample size representing viral isolates from several countries in each respective region.

**Table 2:** Population pair-wise Fst among different regions for genotype 1G.

	AFR	EUR	AMR
AFR	–		
EUR	0.289	–	
AMR	0.072*	0.145	–

Values not significant at *p*<0.01 are indicated with asterisk.

AFR: African region; EUR: European region; AMR: American region.

**Table 3:** Population pair-wise Fst among different regions for genotype 2B.

	WPR	SEAR	EUR	AMR
WPR	–			
SEAR	0.264	–		
EUR	0.202	0.294	–	
AMR	0.172	0.255	0.15	–

WPR: Western Pacific region; SEAR: South East Asian region; EUR: European region; AMR: American region. All values are significant at *p*<0.01.

We did not find statistical evidence for a difference in the evolutionary rate of the two genotypes; however, the upper bound of the 95% HPD for genotype 2B was considerably higher than that of genotype 1G (Table 1). Although the Bayesian plots derived from the E1 sequences of both 1G and 2B genotypes showed a recent decline in the effective number of infections, each genotype showed a unique pattern over time (Fig. 3). GMRF Skyride analyses for genotype 2B showed a recent decline in effective number of infections (Fig. 3B); however, Bayes factor comparisons reveal that demographic histories do not differ significantly from a constant population size model (Table 1). The 1G BSP plot showed an increase in effective number of infections till late-1990s followed by a gradual decline thereafter (Fig. 3A). The very low dN/dS ratio (Table 1), together with no evidence of any positively selected amino acid residues within this functionally important E1 region of the glycoprotein of RV, indicates that both genotypes experienced intense purifying selection, and the emergence/re-emergence of RV 1G and 2B are therefore, unlikely to be vaccine driven. Thus, such dynamic patterns, as revealed for both genotypes, could possibly be associated with the movement of unvaccinated individuals and the extent of the coverage of the MMR vaccination program in respective countries and/or geographic regions.

## Discussion

Recent sporadic rubella outbreaks in Asia, Africa, and South America, as well as the reports of rubella infection in some European countries, remain a global challenge [2], [8]–[10], [13], [15]–[19], [21], [23], [38]–[43]. Understanding the patterns of genetic variation and past population dynamics of this infectious viral pathogen through molecular approaches would significantly contribute towards strategic planning for the elimination of the virus. Employing phylogenetic approaches to whole genome sequence data and to the partial E1 gene sequence data, the present study reports: (1) the timing of emergence of all the thirteen previously described genotypes, (2) the patterns of genetic variations and dispersal patterns of genotypes, 1G and 2B, and (3) the effects of past immunization programs on the population size of these two genotypes.

We estimate the overall substitution rate of RV to be in the order of 10^−3^ subs/site/year, which is compatible with the rate that was previously reported for the 1E genotype of RV [21]. This indicates that the substitution rates of RV are within the compatible range of other aerosol-transmitted human RNA viruses such as measles and mumps that belong to a different family (i.e., Paramyxoviridae) than the arboviruses (e.g., equine encephalitis complexes, Ross River virus, Sindbis virus, Buggy Creek virus, and Highlands J virus) of its own family [44]–[47]. Such remarkably higher substitution rate of RV than the other members of the Togaviridae, which is in the order of 10^−4^ subs/site/year, is a unique characteristic of RV. While RV is a non-vector-borne virus, the other alphaviruses within the same family are arthropod-borne; therefore, vector-host associations are a likely explanation for such discrepancy in the evolutionary rate [44]. Based on the structural comparison of the E1 protein, in a recent landmark discovery, DuBois et al., [48] have shown that the vector-borne viruses belonging to the family Togaviridae are more closely related to the vector-borne viruses in the family Flaviviridae than they are to the RV. This suggests that constraints imposed by the alternative cycles between the human host and arthropod vectors resulted in a more conservative evolution of the vector-borne viruses than the non-vector borne viruses such as RV, which is free from these vector-mediated constraints [48]. The observation of intense purifying selection on the functionally important domain of the E1 glycoprotein, which is characterized by the presence of hemagglutination-inhibiting and -neutralizing epitope and antigenic sites [6], [22], suggests that the RV genotypes 1G and 2B were unlikely to have evolved adaptively, and therefore, indicates the effectiveness of current MMR vaccines used worldwide for the elimination of RV. If these genotypes were evolving adaptively through the modification of the functional epitopes, some amino acid residues within this functionally important E1 protein were expected to be under positive selection [44].

Despite the more than 40 years of vaccination efforts [8]–[11], the spread of RV across the globe, especially in the developing world, warrants detailed investigation on the molecular dynamics of each genotype of RV. Regardless of subtle differences in viral effective population size over time, the BSP plots showed a recent decline, which are consistent with the historical epidemiological trend of the worldwide RV infection since 1970 s and worldwide implementation of immunization programs since 1990 s [11]. Despite the introduction of MMR vaccine in 1970 s, rubella continued to occur in USA and UK until early 1990 s. After the introduction of the second dose of MMR by the United States in 1989, more than 78 countries, mostly from the developed world, adapted national immunization programs by 1996 [11]. By the end of the year, 2002, more than 120 countries/territories across the world implemented mass immunization programs [11]. Thus, the early1990s to ealy-2000s was marked as an important period, during which rubella infections showed a significant decline worldwide. The worldwide immunization programs between the years 1996 to 2002 were consistent with the decline in the effective number of infections as revealed by both genotypes. However, the recent rebound of effective number of infections as shown in genotype 2B is coinciding with the multiple outbreaks reported during 2007–2013 in Southeast Asian countries [14], [17].

Taken together with the conclusions of previous studies, the present study is consistent with the fact that the current vaccines used worldwide for elimination of RV is highly effective; however, the success relies on the extent of vaccine coverage across the regions, especially in the developing world. Further, intercontinental movements of unvaccinated people who are susceptible to rubella infections could also increase the risk of the reappearance of RV and spread of the disease in the regions that have already successfully eliminated the virus. Therefore, effective regulatory measures are recommended to be implemented in order to avoid the spread of this infectious viral pathogen.

## Supporting Information

Appendix S1 **GenBank accession numbers of the sequences used in the analyses.**
(DOCX)
