# Supplementary material for: Molecular Evolutionary and Epidemiological Dynamics of Genotypes 1G and 2B of Rubella Virus
Source: PLoS One. 2014 Oct 17;9(10):e110082. doi: 10.1371/journal.pone.0110082 (PMC4201520; doi:10.1371/journal.pone.0110082)
Supplement: Appendix S1 — GenBank accession numbers of the sequences used in the analyses. (DOCX) [file pone.0110082.s001.docx]

**Supporting Information**

**Accession number of the nucleotide sequences for Figure 1**

AB820865_RVi/Sakai.JPN/11.111j_2011

AY968206_RVI/CAL.USA/97_1997

AY968207_RVI/ISR/75_1975

AY968208_RVI/ISR/79_1979

AY968209_RVI/ISR/88_1988

AY968211_RVI/SLV/02_2002

AY968212_RVI/CAL.USA/91_1991

AY968213_RVI/SHANDONG.CHN/00_1F_2000

AY968215_RVI/Anhui.CHN/00_1F_2000

AY968216_RVI/SAITAMA.JPN/94_1994

AY968217_RVI/PAN/99_1999

DQ085339_Argentina_1988

DQ085340_Russia_1997

DQ085341_Mexico_1997

DQ085342_Korea_1996

DQ085343_Italy_1997

DQ388279_2c_Russia_1967

DQ388280_1G_Germany_1992

DQ388281_NewZealand_1991

DQ454161_Tom9-61.RUS/05_2005

DQ454162_Nvk13-23.RUS/04_2004

EF182759_RVi/Tom9-49.RUS/05_2005

EF182762_RVi/Nsk14-19.RUS/05_2005

EF182765_RVi/Nsk14-74.RUS/06_2006

EF210043_RVs/Jigiga.ETH/09.041g_2004

EF210046_RVs/Guragie.ETH/14.04/21g_2004

EF210050_RVs/London.GBR/52.041gCRS_2004

EF210052_RVs/London.GBR/27.051g_2005

EF672032_RVi/Yilan.TWN/29.05_2005

EU518606_RVs/Madrid.SPA/13.051j_2005

EU518608_RVi/Madrid.SPA/8.05/11j_2005

EU518614_RVs/Madrid.SPA/10.05/31j_2005

EU518617_RVi/Madrid.SPA/42.05/11jCRS_2005

EU518618_RVi/Madrid.SPA/42.05/21jCRS_2005

FJ711663_Rvi/Moscow.RUS/03.08/3_2008

FJ711666_Rvi/Moscow.RUS/15.08_2008

FJ711686_Rvi/Dagestan.RUS/9.08_2008

FJ711687_Rvs/Moscow.RUS/13.07_2007

FJ711689_Rvs/Krasnoyarsk.RUS/17.04/2_2004

FJ711690_Rvs/Chelyabinsk.RUS/13.08/5_2008

FJ711693_Rvi/Kursk.RUS/8.08/2_2008

FJ711694_Rvs/Tula.RUS/06.06_2006

FJ711696_Rvi/Ryazan.RUS/09.08_2008

FJ711698_Rvs/Krasnoyarsk.RUS/17.04/3_2004

FJ774999_RVi/Deweim.SDN/50.051GCRS_2005

FJ875030_RVi/Zibo.Shandong.CHN/40.01/1_2001

FJ875031_RVi/Zibo.Shandong.CHN/40.01/2_2001

FJ875032_RVi/Jining.Shandong.CHN/40.01_2001

FJ875035_RVi/Mengcheng.Anhui.CHN/20.99/5_1F_1999

FJ875046_RVi/Xuchang.Henan.CHN/13.02/1_1F_2002

FN546968_RVs/Lille.FRA/38.951h_1995

FN546969_RVs/Paris.FRA/18.961G_1996

FN546985_RVs/Marseille.FRA/17.981G_1998

FN547005_RVs/Paris.FRA/35.011BCRS_France_1B_2001

GQ329848_RVs/RioGrandedoSul.BRA/48.05_2005

GQ329849_RVs/RioGrandedoSul.BRA/49.05_2005

GQ329850_RVs/RioGrandedoSul.BRA/50.05_2005

GU053583_RVi/SaoPaulo02.BRA/99_Brazil_1a_1999

GU968187_RVi/SaoPauloBRA/30.07_Brazil_1a_2007

HM461998_RVs/HongKong.CHN/18.10_2010

JN575762_RVs/BritishColumbia.CAN/25.11/1_2011

JN635281_1a_USA_1961

JN635282_1B_Venezuela_1998

JN635283_RVi/LA.CA.USA/91/1C_1991

JN635284_RVi/TX.USA/34.98/1C_1998

JN635285_RVi/CA.USA/88/1D_1988

JN635286_1E_USA__Maine_2008

JN635287_1E_Ukraine_1998

JN635288_1E_China_2008

JN635289_1G_Uganda_2007

JN635290_1G_CIV_2005

JN635291_RVi/Daly_City.CA.USA/97/1j_CRS_1997

JN635292_2B_USA_2007

JN635293_2B_India_2000

JN635294_2B_India_2008

JN635295_2B_USA_Minnesota_2009

JN635296_2B_India_2008

JQ900444_RVs/Taipei.TWN/17.05_2005

JQ900445_RVs/Kaohsiung.TWN/20.05_2005

JQ900446_RVs/Hsinchu.TWN/48.08_2008

JQ900447_RVs/Taichung.TWN/44.11_2011

JX398312_UK_2012

JX477653_Miami.FL.USA/21.10_2010

JX477654_Yavapai.AZ.USA/4.10_2010

JX477655_Baltimore.MD.USA/6.12_2012

JX477656_Birmingham.AL.USA/11.12_2012

JX546595_Rvi/Sao_PauloBRA/42/09_CRI_Brazil_1a_2009

JX679257_RVS/LimaSur/Peru/1.05/1C_2005

JX679260_RVS/Lima.Peru/51.04-1/1C_2004

JX679261_RVS/Lima.PER/50.04/1C_2004

JX679272_CotedIvoire_2008

JX679273_Ghana_2008

JX679274_Ghana_2005

JX679276_Ghana_2005

JX679277_RVs/BLZ/6.94/1C_1994

JX679278_RVs/HND/12.00/1C_2000

JX679280_RVs/LimaSur.PER/2.05/1C_2005

KC759166_RVs/HongKong.CHN/07.132A_2007

KC884222_RVi/Kiboga.UGA/13.03_2003

KC884223_RVi/Kiboga.UGA/18.03_2003

KC884225_RVi/Kasese.UGA/21.08/2_2008

KC884227_RVi/Oyam.UGA/35.09_2009

KC884228_RVi/Jinja.UGA/50.10_2010

KC884229_RVi/Jinja.UGA/52.10/1_2010

KC884231_RVi/Rakai.UGA/05.11_2011

KC884238_RVi/Mityana.UGA/50.11_2011

KC884239_RVi/Lwengo.UGA/10.12_2012

KC884240_RVi/Kiryandongo.UGA/11.12_2012

KC962556_Brazil_2003

KC962558_Brazil_2004

KF201674_1E_China_2002

KF792832_RVi/London.GBR/86/1I_1986

KF792833_RVi/Milan.ITA/46.92/1I_1992

AY326335_Israel_1968

AY326349_China_1979

**Accession number of nucleotide sequences used for genotype 1G analysis**

KC884227_Uganda__2009

AY161364_Italy_1993

AY161366_Italy_1994

AY161367_Italy_1994

DQ388280_Germany_1992

AF039130_UK_1993

AY161361_Italy_1993

KC962556_Brazil_2003

KC962558_Brazil_2004

FJ711662_Russia_2008

FJ711664_Russia_2008

EF210052_UK_2005

FJ711665_Russia_2008

FJ711660_Russia_2006

FJ711663_Russia_2008

FJ711661_Russia_2008

FJ711666_Russia_2008

AM258945_Belarus_2004

AM258952_Belarus_2004

AM258949_Belarus_2005

AM258950_Belarus_2004

AM258951_Belarus_2004

AM258944_Belarus_2005

EF588970_Canada_2005

AF039133_Germany_1995

AY326342_Germany_1998

AY161365_Italy_1994

AY161368_Italy_1994

AF039128_UK_1993

AF039129_UK_1993

FN546969_France_1996

AF039123_uk_1991

AY326341_Germany_1991

AF039131_UK_1993

AY326340_Israel_1992

AY161371_Italy_1995

AY161372_Italy_1995

AY161373_Italy_1995

FN546985_France_1998

JX477654_USA_Kenya_2010

EF210043_Ethiopia_2004

EF210044_Ethiopia_2004

EF210045_Ethiopia_2004

EF210046_Ethiopia_2004

JQ627831_Portugal_2009

EF588979_CotedIvoire_2005

JN635290_CotedIvoire_2005

JX477656_USA_Nigeria_2012

JX679266_CotedIvoire_2008

JX679267_CotedIvoire_2008

JX679273_Ghana_2008

JX679275_Ghana_2004

JX679276_Ghana_2005

JX679274_Ghana_2005

JX679265_CotedIvoire_2008

JX679268_CotedIvoire_2008

JX679269_CotedIvoire_2008

JX679272_CotedIvoire_2008

JX679271_CotedIvoire_2008

JX679270_CotedIvoire_2008

FJ774999_Sudan_2005

EF588978_Uganda_2001

KC884223_Uganda__2003

JX477655_USA_Tanzania_2012

KC884222_Uganda__2003

KC884224_Uganda__2008

KC884225_Uganda__2008

KC884239_Uganda__2012

KC884228_Uganda__2010

KC884229_Uganda__2010

KC884230_Uganda__2010

KC884240_Uganda_2012

KC884235_Uganda__2011

KC884236_Uganda__2011

KC884234_Uganda__2011

KC884231_Uganda__2011

JX398310_UK_2012

JX398311_UK_2012

JX398312_UK_2012

KC884232_Uganda__2011

KC884233_Uganda__2011

KC884237_Uganda__2011

KC884238_Uganda__2011

EF588977_USA_Uganda_2007

JN635289_USA_Uganda_2007

EF210050_UK_2004

KC884226_Uganda_2009

**Accession number of the nucleotide sequences used for Genotype 2B analyses**

AY326345_Korea_1995

AB706304_Vietnam_2011

AB706303_Vietnam_2011

AB745031_Vietnam_2011

JQ900418_Taiwan_2011

HQ893753_Vietnam_2010

FJ656218_HongKong_2008

JN661172_Malaysia_2011

JN661171_Malaysia_2011

AB702681_Japan_2011

JQ900424_Taiwan_2011

JQ900423_Taiwan_2011

AB706307_Vietnam_2011

AB706306_Vietnam_2011

JQ900404_Taiwan_2011

AB735187_Japan_2012

JX398308_UK_2012

AB745037_Vietnam_2011

AB745032_Vietnam_2011

AB706308_Vietnam_2011

AB706300_Vietnam_2011

HQ893754_Vietnam_2010

JQ900416_Taiwan_2011

AB706305_Vietnam_2011

AB845509_Japan_2012

JQ900429_Vietnam_2011

JX398301_UK_2011

AB745028_Vietnam_2011

JN827384_HongKong_2011

KC917271_HongKong_China_2011

KF792830_Hawaii_USA_Japan_2013

KF305760_HongKong_China_2013

AB845520_Japan_2013

AB753258_Japan_2012

AB897699_Japan_2013

AB845521_Japan_2013

AB824848_Japan_2012

AB753259_Japan_2012

AB830113_Japan_2013

AB824851_Japan_2012

AB824850_Japan_2012

JQ900406_Taiwan_2011

JQ900403_Taiwan_2011

AB706302_Vietnam_2011

AB706298_Vietnam_2011

HQ893756_Vietnam_2010

HQ893757_Vietnam_2010

HQ893750_Vietnam_2010

HQ893755_Vietnam_2010

HQ893758_Vietnam_2010

JQ900417_Taiwan_2011

AB706301_Vietnam_2011

JQ900430_Taiwan_2011

JQ900421_Taiwan_2011

JQ900409_Taiwan_2011

AB706299_Vietnam_2011

JX036508_HongKong_2012

JQ031213_HongKong_2011

AB632389_Japan_2011

JF911797_Canada_2011

AB665169_Japan_2011

HQ893751_Vietnam_2010

AB702686_Japan_2012

AB702685_Japan_2011

AB702684_Japan_2011

AB702682_Japan_2011

AB702680_Japan_2011

JQ900438_Taiwan_2009

JN661170_Malaysia_2011

JN661165_Malaysia_2011

JN661169_Malaysia_2011

JN661168_Malaysia_2011

JN661164_Malaysia_2011

JN661166_Malaysia_2011

JN661167_Malaysia_2011

JN661163_Malaysia_2011

FJ971783_Argentina_2008

FJ971778_Argentina_2008

FJ971771_Argentina_2008

FJ971770_Argentina_2008

FJ971776_Argentina_2008

FJ971769_Argentina_2008

FJ971779_Argentina_2008

FJ971762_Argentina_2008

FJ971774_Argentina_2008

FJ971777_Argentina_2008

FJ971780_Argentina_2008

FJ971763_Argentina_2008

JN582037_Argentina_2009

FJ971782_Argentina_2008

FJ971775_Argentina_2008

FJ971773_Argentina_2008

FJ971767_Argentina_2008

FJ971772_Argentina_2008

FJ971766_Argentina_2008

FJ971761_Argentina_2008

JN582035_Argentina_2009

JN582036_Argentina_2009

FJ971768_Argentina_2008

FJ971764_Argentina_2008

HM212632_Brazil_2009

FJ971765_Argentina_2008

EU240900_UK_2007

GU254254_Brazil_2008

HM212631_Brazil_2008

HM212630_Brazil_2008

HM212634_Brazil_2009

FJ971781_Argentina_2008

GQ374572_China_2008

GU254251_Brazil_2006

FR717221_Bosnia-Herzegovina_2010

FR717220_Bosnia-Herzegovina_2010

FR717213_Bosnia-Herzegovina_2010

FR717212_Bosnia-Herzegovina_2010

FR717210_Bosnia-Herzegovina_2010

FR717209_Bosnia-Herzegovina_2009

FR717222_Bosnia-Herzegovina_2010

FR717218_Bosnia-Herzegovina_2010

FR717214_Bosnia-Herzegovina_2010

FN547021_France_2009

FR717211_Bosnia-Herzegovina_2010

FR717206_Bosnia-Herzegovina_2009

FR717207_Bosnia-Herzegovina_2009

FR717208_Bosnia-Herzegovina_2009

FR717216_Bosnia-Herzegovina_2010

FR717217_Bosnia-Herzegovina_2010

FR717219_Bosnia-Herzegovina_2010

FR717215_Bosnia-Herzegovina_2010

GU254255_Brazil_2008

GU254253_Brazil_2007

JN635294_USA_India_2008

GU353074_USA_India_2009

HM212633_Brazil_2008

GU254252_Brazil_2007

JQ283994_India_2007

FJ656219_HongKong_2008

JQ283993_India_2007

JQ283995_India_2008

JN635295_USA_2009

GU353075_USA_2009

GU353072_USA_Mexico_2008

FN547017_France_2004

EU240899_UK_2007

JQ413980_India_2009

JN635296_USA_India_2008

GU353073_USA_India_2008

AB674470_Japan_2011

GU174756_Canada_2009

JN635293_USA_India_2000

AY968220_USA_2000

AY326343_India_2000

FJ711685_Kazakhstan_2008

JN635292_USA_2007

GU353071_USA_2007

GU289731_China_2009

GU289729_China_2009

AB546233_Vietnam_2009

FJ875057_China_2006

HQ893752_Vietnam_2009

HQ893749_Vietnam_2010

AY161370_Italy_1994

AF039134_India_1995

AY326335_Israel_1968

JF702871_China_2008

JF702870_China_2008

AY968218_China_2000

DQ085342_Korea_1996

AY326347_Korea_1996

**Accession number of the nucleotide sequences used for whole genome analyses**

JN635281_USA_1961

JN635282_Venezuela_1998

JN635283_USA_1991

JN635284_USA_1998

JN635285_USA_1988

JN635287_Ukraine_1998

KF201674_China_2002

JN635288_China_2008

JN635286_USA_Maine_2008

JN635290_CIV_2005

JN635289_Uganda_2007

DQ388280_Germany_1992

JN635291_USA_1997

JN635293_India_2000

JN635296_India_2008

JN635294_India_2008

JN635292_USA_2007

JN635295_USA_Minnesota_2009

DQ388279_Russia_1967

DQ085339_Argentina_1988

DQ388281_NewZealand_1991

DQ085343_Italy_1997

DQ085341_Mexico_1997

DQ085340_Russia_1997

DQ085338_Israel_1968

DQ085342_Korea_1996

AY258322_China_1979
